# Obesity is a negative predictor of success after surgery for complex anal fistula

**DOI:** 10.1186/1471-230X-11-61

**Published:** 2011-05-23

**Authors:** O Schwandner

**Affiliations:** 1 Department of Proctology, Krankenhaus Barmherzige Brueder Regensburg, Regensburg, Germany

**Keywords:** Anal fistula, obesity, success, recurrence

## Abstract

**Background:**

It was the aim of this study to compare the outcome of surgery for complex anal fistulas in obese and non-obese patients.

**Methods:**

All patients with complex anorectal fistulas who underwent fistulectomy and/or rectal advancement flap repair were prospectively recorded. Surgery was performed in a standardized technique. Body mass index (BMI [kg/m^2^]) was used as objective measure to indicate morbid obesity. Patients with a BMI greater than 30 were defined as obese, and patients with a BMI below 30 were defined as non-obese. The parameters analyzed related to BMI included success or failure, and reoperation rate due to recurrent abscess. Success was defined as closure of both internal and external openings, absence of drainage without further intervention, and absence of abscess formation.

**Results:**

Within two years, 220 patients underwent advancement flap repair and met the inclusion criteria. 55% of patients were females, mean age was 39 (range 18-76) years, and the majority of fistulas were located at the posterior site. 69% of patients (152/220) were non-obese (BMI < 30), whereas 31% (68/220) were obese (BMI > 30). After a median follow-up of 6 months, primary healing rate ("success") for the whole collective was 82% (180/220). Success was significantly different between non-obese and obese patients: In non-obese patients, recurrence rate was significantly lower than in obese patients (14% vs. 28%; p < 0.01). Moreover, reoperation rate due to recurrent abscess with the need for seton drainage in the failure groups was significantly higher in obese patients when compared to non-obese patients (73% vs. 52%; p < 0.01). Using multivariate analysis, obesity was identified as independent predictive factor of success or failure (p < 0.02).

**Conclusion:**

Obese patients are at higher risk for failure after surgery for complex anal fistula.

## Background

The main issue of surgery for anal fistulas is to provide definite healing and to prevent incontinence. Focussing on complex anal fistulas, it can be stated that rectal advancement flap repair represents an effective treatment option [1,2]. To date, neither the addition of fibrin glue or local administration of antibiotics nor the use of bioprosthetic plugs did improve healing rates when compared to advancement flap repair alone [2-8]. The main advantages of the advancement flap repair are that it is repeatable, and that the flap procedure can be combined with sphincter reconstruction in anterior fistula which has a tremendous impact on continence especially in females. However, although the sphincter muscle is not divided during the flap repair, minor impairement of continence has been reported in up to a third and major incontinence in approximately 5-10% of patients. Additionally, looking for predictors of success or failure, undrained perianal sepsis, complexity of fistula, missing identification of the internal opening, number of previous attempts to close the fistula, Crohn's disease, rectovaginal fistula, and previous radiation therapy have been shown to deteriorate primary success rates [2].

At present, obesity is one of the most important health problem and it is known to aggravate numerous health disorders such as cardiovascular disease, hypertension, and metabolic disorders. Focussing on the potential of comorbid conditions, therefore, obese patients are generally believed to be at increased risk for surgery or adverse outcomes than those who are not obese [9]. However, to date, there is no information available, whether obesity represents a factor predictive of failure or recurrence following definite surgery for anal fistula of cryptoglandular origin. Therefore, it was the aim of this prospective study to compare the outcome of fistulectomy and rectal advancement flap repair for complex anal fistulas in obese and non-obese patients.

## Methods

### Study design

For the current study, the body mass index (BMI [kg/m^2^]) was used as objective measure to indicate morbid obesity because it fulfils the mandatory criteria for an epidemiological index of obesity [9]. Patients with a BMI greater than 30 (> or = 30) were defined as obese, and patients with a BMI below 30 (<30) were defined as non-obese.

All patients with complex anal fistulas who underwent a definite surgical closure performed by one surgeon (fistulectomy, rectal advancement flap repair) were prospectively enrolled, and data were retrospectively analyzed in relation to obesity. The study was conducted at the Caritas-Krankenhaus St. Josef Regensburg. As the study was conducted as a retrospective observational outcome analysis without any experimental research, ethical commitee approval was not necessary. Inclusion criteria included supra-, trans- or intersphincteric fistulas of cryptoglandular origin according to Parks classification [10]. To provide a homogenous patient collective, rectovaginal and Crohn's associated anal fistula as well as patients with fecal diversion were excluded from analysis. Furthermore, patients in which the internal opening could not be identified during primary surgery were excluded (Table 1).

**Table 1:** Inclusion and exclusion criteria

Inclusion criteria	Exclusion criteria
Transsphincteric fistula of cryptoglandular origin	Superficial fistulas amenable for fistulotomy

Suprasphincteric fistula of cryptoglandular origin	Rectovaginal and fistulas associated to Crohn's disease

High intersphincteric fistula of cryptoglandular origin	Concomitant anorectal abscess or internal opening not identified

Seton drainage	Fecal diversion

Primary end-points of this study included success or failure related to body status. Success was defined as closure of both internal and external openings, absence of drainage without further intervention, and absence of abscess formation. Additionally, morbidity and reoperation rate for abscess recurrence were analyzed. In all patients, informed consent was obtained. The study was self-funded, and no financial support was received.

### Surgical Technique

All patients were examined preoperatively in the proctological office to exclude residual anorectal abscess. Patients who had seton drainage due to previous drainage of concomitant abscess were examined with endoanal ultrasound. MRI was not used routinely. Focussing on surgical strategy, patients who had seton drainage were scheduled for definite surgery after a time interval of 6-8 weeks. In addition to fistulectomy, fistulas in which the fistula tract was located in the upper two-thirds of the external sphincter were treated by rectal advancement flap repair. On the day of surgery, bowel preparation was performed by enema, no patient underwent mechanical bowel preparation. A single-dose antibiotic prophylaxis (cefotaxime and metronidazole) was mandatory. Procedures were either performed under general or spinal anesthesia (according to patients' preference) and in lithotomy position.

All fistula tracts (including internal and external opening) were identified with a conventional fistula probe in all patients (with or without a seton drainage) to define the type of fistula according to the Parks classification [10]. After replacement of the seton drainage, the fistula tract was irrigated with antiseptic polyhexanide solution (Lavanid^®^1; Serag Wiessner, Naila/Bayern, Germany). It was crucial to excise the fistula tract running from the external opening to the external anal sphincter and to have a sufficient external drainage wound. Using a Parks or Simms retractor, a full-thickness flap consisting of mucosa, submucosa, and the internal sphincter was mobilized from the level of the dentate line to 2-6 cm cephalad. The base of the flap was approximately twice the width of its apex. The crypt-bearing tissue around the internal opening was excised, and the fistula tract was cored out of the sphincters. The defect of the internal anal sphincter was closed with absorbable sutures (Vicryl 2/0^®^, Ethicon Endo-Surgery, Norderstedt, Germany). After excision of its apex, the full-thickness flap is advanced and sutured without any tension to the "neodentate line" or to the subcutaneous parts of the external sphincter where appropriate using absorbable sutures (Vicryl 3/0^®^, Ethicon Endo-Surgery, Norderstedt, Germany).

Postoperatively, immediate feeding (regular food) was administered, no further antibiotics were given. Patients were discharged on the 1st postoperative day.

### Follow-up

All patients were followed up within 2-4 weeks after surgery to monitor regular wound healing. Specific follow-up information was derived from clinical examination 3 and 12 months postoperatively; moreover, time intervals of patients with recurrence or failure were registered. The follow-up was performed in an office setting with regular clinical examination (including digital examination, proctoscopy, evaluation of the fistula status by using a conventional fistula probe) and assessment of success or failure. Follow-up examination was performed by the operating surgeon (O.S.).

### Statistical analysis

Statistics included both univariate and multivariate analysis for comparison of success rates using the SPSS^® ^software package. Only factors which reached statistical significance in univariate analysis were entered into multivariate analysis. BMI was analyzed as a dichotomous variable to provide sufficient patient numbers. Statistical significance was assessed at the 5% level (p < 0.05 statistically significant).

## Results

Between September 2007 and September 2009, 220 patients were operated on for anal fistulas in the study period and met the inclusion criteria. 55% of patients were females, mean age was 39 (range 18-76) years, and the majority of fistulas were located at the posterior site. Details of patients' demographics, fistulas, co-morbidities, and type of surgery are summarized in Table 2. 69% of patients (152/220) were non-obese (BMI < 30), whereas 31% (68/220) were obese (BMI > 30). The majority of patients underwent rectal advancement flap repair, whereas a minority had fistulectomy (Table 2). Postoperative morbidity occurred in 6 patients (minor bleeding 1; urinary tract infection 1; urinary retention 1; sphincter spasm with pain 3) regardless of body-mass status. After a median follow-up of 6 (range 3-18) months, primary healing rate ("success") for the whole collective was 82% (180/220). As outlined in Table 3, success was significantly different between non-obese and obese patients: In non-obese patients, recurrence rate was significantly lower (14%; 21/152) than in obese patients (28%; 19/68) (p < 0.01). Moreover, reoperation rate due to recurrent abscess with the need for seton drainage in the failure groups was significantly higher in obese patients when compared to non-obese patients (73%; 14/19 vs. 52%; 11/21) (p < 0.01). No other factor (age, tertiary referral, fistula location, number of previous attempts to close the fistula, smoking, diabetes, type of surgery) was associated with outcome. Using multivariate regression analysis, obesity was identified as independent predictive factor of success or failure (p < 0.02, multivariate hazard ratio 3.35).

**Table 2:** Results: Patients'demographics, fistulas and surgical data

Variable	BMI < 30	BMI ≥ 30
No. of patients	152	68

Mean age (years)	39 (21-76)	39 (18-64)

Tertiary referral (%)	46 (30%)	22 (32%)

Previous fistula surgery prior to advancement flap		
0	118 (78%)	40 (58%)
1	12 (8%)	11 (16%)
2	10 (6%)	9 (13%)
3 or more	12 (8%)	8 (13%)

Fistula type		
transsphincteric	96 (63%)	45 (66%)
intersphincteric	50 (33%)	19 (28%)
suprasphincteric	6 (4%)	4 (6%)

Fistula location		
anterior	32 (21%)	10 (15%)
posterior	120 (79%)	58 (85%)

Horse-shoe-fistula (%)	6 (4%)	5 (7%)

Seton drainage (%)	132 (87%)	58 (85%)

Co-morbidity (cardiovascular, diabetes)	30 (20%)	30 (44%)

Co-morbidity (smoking)	50 (33%)	25 (37%)

Advancement flap repair (%)	108 (71%)	47 (69%)

Mean operative time (min)	20 (12-32)	24 (18-40)

Morbidity (%)	3 (2%)	3 (4%)

**Table 3:** Success and failure rates

Variable	BMI < 30	BMI ≥ 30
Overall success rate	86% (131/152)	72% (49/68)

Overall failure rate	14% (21/152)	28% (19/68)

Reoperation rate due to recurrent abscess	52% (11/21)	73% (14/19)

## Discussion

Objectively, there is only little evidence on the "optimal" treatment for complex anal fistulas. A systematic review published by Malik and Nelson identified 21 randomized studies and 2 meta-analyses for evaluation [2]. The only conclusions which could be derived from the literature were that flap repair may not be worse than fistulotomy related to healing rates, and that flap repair in combination with fibrin glue may increase failure rates [2]. Following this review, only 3 randomized studies were available to assess the impact of rectal advancement flap repair [11-13]. In general, two of these randomized compared advancement flap repair with "conventional" treatment (e.g. fistulotomy) [11,12], whereas Gustaffson and colleagues examined whether the addition of an antibiotic impregnated sponge would influence success [13]. In conclusion of these studies, there were neither significant differences in healing rates nor differences in postoperative continence status [11,12]. Moreover, the addition of local antibiotics did not improve the outcome [13].

Based on the course of the fistula, all complex fistula including transsphincteric, suprasphincteric, and high intersphincteric fistulas not amenable for fistulotomy are potential indications for a flap repair. It is a prerequisite that perianal sepsis has been eradicated previously (e.g. excision of abscess with seton drainage of corresponding fistula). From the technical aspect, fistulectomy with removal of all fistula tracts may be a crucial factor, and a sufficient external wound is mandatory.

The key question of surgery for complex anal fistulas is whether factors predictive of success or failure can be identified. Based on the literature, undrained perianal sepsis, recurrent fistula, Crohn's disease, rectovaginal fistula, smoking, and previous radiation therapy have been shown to deteriorate outcome [14-19].

In the 90ies, Schouten and colleagues demonstrated clearly that the success rate was inversely correlated with the number of prior attempts to close the fistula [14]: Transrectal advancement flap repair for transsphincteric fistula showed a success rate of 75% in total; however, if flap repair was related to the number of prior attempts, the authors indicated a 87% success rate (no or only one previous repair) versus a 50% success rate (two or more previous attempts) [14]. Additionally, a high success rate of 75% for the whole collective was combined with a 35% rate of deterioration of continence status [14].

Mizrahi and colleagues reported a success rate of 59.6% (after a mean follow-up of 40 months) after flap repair for both cryptoglandular and Crohn's associated fistulas [15]. Conversely, prior attempts to close the fistula did not negatively influence outcome [15]. As expected, a significantly higher recurrence rate (57.1%) was observed after flap repair for complex fistulas associated with Crohn's disease when compared to fistulas without Crohn's disease (33.3%) [15].

Ellis demonstrated that the risk of failure was associated with a history of previous attempts of fistula repair and tobacco smoking [16].

A Dutch series with extended follow-up (median follow-up 76 months) published by van Koperen and colleagues identified risk factors for recurrence after advancement flap repair: However, they did not find any - neither prior fistula surgery, smoking nor fibrin glue [17].

An Austrian group assessed the influence of the type of flap repair. They compared classical advancement flap with mucosal flap in a series of 54 patients with high transsphincteric fistula. They clearly demonstrated that the advancement flap representing a full-thickness flap was associated with a highly significant better outcome without any negative impact on continence [18].

Moreover, the study of Mitalas and colleagues impressively showed that the flap is repeatable. Reporting on 87 rectal advancement flap procedures, the primary healing rate was 67%. In these patients who had primary failure and underwent "repeat flap", the healing rate was 69%. Altogether, a 90% overall healing rate could be achieved without any negative impact on continence [19].

Summarizing the majority of available studies, it becomes obvious that the management of complex fistulas is challenging, that clear conclusions based on these studies cannot be drawn, and, that many gaps related to primary failure or recurrence still remain.

At present, obesity is one of the most challenging health problem in industrialized states with tremendous impact on health systems and financial ressources. Obesity is known to predispose for numerous health disorders, and has been shown to be one of the most important risk factor predictive of the development of metabolic, endocrine and cardiovascular pathologies such as diabetes, hypertension and coronary heart disease. Moreover, health consequences and co-morbidities of obesity may negatively influence quality of life and life-expectancy itself. As it has been proven that obese patients have a higher incidence of comorbid conditions, it has been proposed that these patients are at higher risk of morbidity (e.g. wound infections, cardiopulmonary complications) after surgery. Based on these concepts, obese patients are generally believed to be at a higher risk for surgery than those patients who are not obese, although evidence-based convincing data are lacking. Consequently, a variety of studies specifically focussed on the impact of obesity on surgical outcomes. However, there are no data available focussing on the role of obesity on success or failure rates in surgery for complex anal fistulas.

The current results indicate that obese patients suffering from complex anal fistulas had a worse outcome related to success. Moreover, obesity was identified as independent predictive factor for failure - and this was not related to a higher incidence of co-morbidities such as diabetes in obese patients. Additionally, reoperation rate with the need for seton drainage due to recurrent perianal abscess was significantly higher when compared to non-obese patients. It can only be speculated which reasons were associated with these findings. It can be stated that the prevalence of co-morbidities was significantly increased in obese patients when compared to non-obese patients. Potentially, the local wound healing of the flap was compromized in this subgroup of obese patients. This would be in accordance to the role of smoking: Zimmerman and colleagues demonstrated that smoking was associated with a higher risk for fistula recurrence after advancement flap repair [20]. Another reason could be that local hypoperfusion in fat tissue negatively influences local wound healing. However, a generally accepted cause for the presented results cannot be provided and reasons remain speculative. Certainly, one has to admit that the study has some limitations including a short follow-up period, a potential reporting bias, and no evaluation of continence data.

## Conclusion

Obese patients are at higher risk to develop failure or recurrence after surgery for complex anal fistulas.

## Competing interests

The authors declare that they have no competing interests.

## Pre-publication history

The pre-publication history for this paper can be accessed here:

http://www.biomedcentral.com/1471-230X/11/61/prepub
